# The role of APC/C in cell cycle dynamics, growth and development in cereal crops

**DOI:** 10.3389/fpls.2022.987919

**Published:** 2022-09-29

**Authors:** Perla Novais de Oliveira, Luís Felipe Correa da Silva, Nubia Barbosa Eloy

**Affiliations:** Department of Biological Sciences, Escola Superior de Agricultura ‘Luiz de Queiroz’, University of São Paulo, Piracicaba, Brazil

**Keywords:** cell cycle, cereal crops, plant development, anaphase promoting complex/cyclosome, plant growth

## Abstract

Cereal crops can be considered the basis of human civilization. Thus, it is not surprising that these crops are grown in larger quantities worldwide than any other food supply and provide more energy to humankind than any other provision. Additionally, attempts to harness biomass consumption continue to increase to meet human energy needs. The high pressures for energy will determine the demand for crop plants as resources for biofuel, heat, and electricity. Thus, the search for plant traits associated with genetic increases in yield is mandatory. In multicellular organisms, including plants, growth and development are driven by cell division. These processes require a sequence of intricated events that are carried out by various protein complexes and molecules that act punctually throughout the cycle. Temporal controlled degradation of key cell division proteins ensures a correct onset of the different cell cycle phases and exit from the cell division program. Considering the cell cycle, the Anaphase-Promoting Complex/Cyclosome (APC/C) is an important conserved multi-subunit ubiquitin ligase, marking targets for degradation by the 26S proteasome. Studies on plant APC/C subunits and activators, mainly in the model plant Arabidopsis, revealed that they play a pivotal role in several developmental processes during growth. However, little is known about the role of APC/C in cereal crops. Here, we discuss the current understanding of the APC/C controlling cereal crop development.

## Introduction

Monocotyledon crops, such as maize, rice, sorghum, wheat, and sugarcane, have a huge impact on different aspects of human society, such as feed and food supply and biofuel production, being the basis of the economy in several countries. Since the implementation of monocultures in agriculture, humankind developed strategies to increase crop yield. Through the domestication of crop species, farmers began to identify individuals with better traits among others of the same species present in the plantation and started to selectively propagate them for the next harvest. A large part of the cultivated varieties grown nowadays was produced by this technique, after the theoretical framework provided by Gregor Mendel with the establishment of the heredity principles (Moose and Mumm, 2008; Tester and Langridge, 2010). The following years were marked by the definition of genetic inheritance laws and advances in molecular biology (Kellenberger, 2004). These events have paved the way for more detailed studies on key individual components that affect specific plant characteristics, such as life cycle, hormonal response, growth and development. This technical-scientific revolution allowed the emergence of new techniques, including genetically modified plants, to increase agricultural productivity, highlighting the importance of characterizing basic biological processes (Botstein, 2012).

Growth and development are two well-characterized processes extensively studied in plants. The first refers to the permanent and irreversible increase in volume and biomass of the plant, which may or may not be accompanied by the addition of new organs (Brukhin and Morozova, 2011). Development, in turn, is responsible for the physical and morphological changes in the plant body throughout its different stages of life (Drost et al., 2017). Those two processes depend on energetic reactions that generate specific cell patterns, which form specialized tissues and shape the plant organs. Most plants exhibit an indeterminate growth pattern, being able to grow even after reaching reproductive maturity, an ability that differs from most animals that achieve a maximum size at a specific age (Brukhin and Morozova, 2011; Perianez-Rodriguez et al., 2014; Hariharan et al., 2016). This indeterminate growth is due to the continuous activity of meristematic tissues, allowing the generation of new plant organs. The cells in these meristematic tissues divide and generate new cells, some will remain as meristematic cells while others will undergo differentiation and specialization, becoming derivative cells. The specialized cells ensure that each organ will play the function that is fated after cellular specialization, the new cells continue to divide for some time to propagate the differentiated region (Doerner, 2003; Stahl and Simon, 2010; Hariharan et al., 2016; Kitagawa and Jackson, 2019; Umeda et al., 2021). Thus, the development of an organism comprises a set of processes that allow the transition from single cells to a complex multicellular organism. Most animals, except for species that undergo metamorphosis, complete their ontogenetic development during embryogenesis, so the body plan of the mature embryo is extremely similar to the adult but in smaller proportions. Conversely, plants do not have an endpoint for ontogenetic development, even after the short embryonic development phase (Hariharan et al., 2016; Drost et al., 2017);. Throughout embryogenesis, meristems originate only primary structures, such as hypocotyl, cotyledons, and radicle (de Vries and Weijers, 2017; Radoeva et al., 2019; Armenta-Medina et al., 2020). Most of the true tissues and organs, including flowers, roots, stems, and vascular systems, develop after seed germination, in a post-embryonic developmental program, which, like growth, occurs throughout the lifespan of the plant. Moreover, organ development in plants occurs in a sequentially way, by the addition of functional units called phytomers (McMaster, 2005; Javelle et al., 2011; Perianez-Rodriguez et al., 2014).

## Cell division

In multicellular organisms, including plants, the processes of growth and development are driven by cell division (Sablowski and Carnier, 2014). The cell cycle brings together different molecular and biochemical events that allow the emergence of new cells (Inzé and De Veylder, 2006). Cell division is characterized by four sequential phases, which temporally separate the replication of genetic material from the segregation of homologous chromosomes into two daughter cells, making up the mitotic cell cycle. The DNA replication (S) and mitotic entry (M) phases are separated by two gap (G) phases. The G1 phase separates the end of mitosis and the sequential S phase, while the G2 phase precedes the entry of mitosis after the end of the S phase. Thus, cells in G2 have twice the genetic material compared to cells in G1. G phases have molecular mechanisms capable of verifying whether the previous phase was completed correctly (Hunt et al., 2011; Kernan et al., 2018; Matthews et al., 2022).The process of cell division requires a sequence of intricated events that are carried out by a variety of protein complexes and molecules that act punctually throughout the cycle.

In all eukariotes, including plants, the cell cycle progression relies on the activity of the CDKs (cyclin-dependent kinases), its activity is essential to trigger the transition from the G_1_ to S and the G_2_ to M phases. CDK regulation happens through association with its regulatory subunits known as cyclins, phosphorylation, dephosphorylation, interaction with inhibitory proteins, and proteolysis (de Veylder et al., 2007). However, the primary process which control the cell cycle evolution are similar in all eukariotes, plants has unique features controlling it. Usually they possess many more CYCs and CDKs compared with yeast and animals. For example, in the Arabidopsis genome there are 7 classes of Cyclins, comprising about 50 genes, some of which have unknown functions. Among them, the most studied are the A, B and D classes (Shimotohno et al., 2021).The A-type cyclins are the regulators of S to M transition, B-type cyclins control the G2 to M transition, while D-type Cyclins are the G1-S trasition regulators, thus specific interactions between different CYCs and CDKs are the key feature to recognize the targets and promote regulation of the differents cell cycle phases. About CDKs, the most known are the CDKAs and CDKBs, being the latter only found in the plant kingdom, which are directly involved in cell cycle control (Polyn et al., 2015).

**Figure 1:**
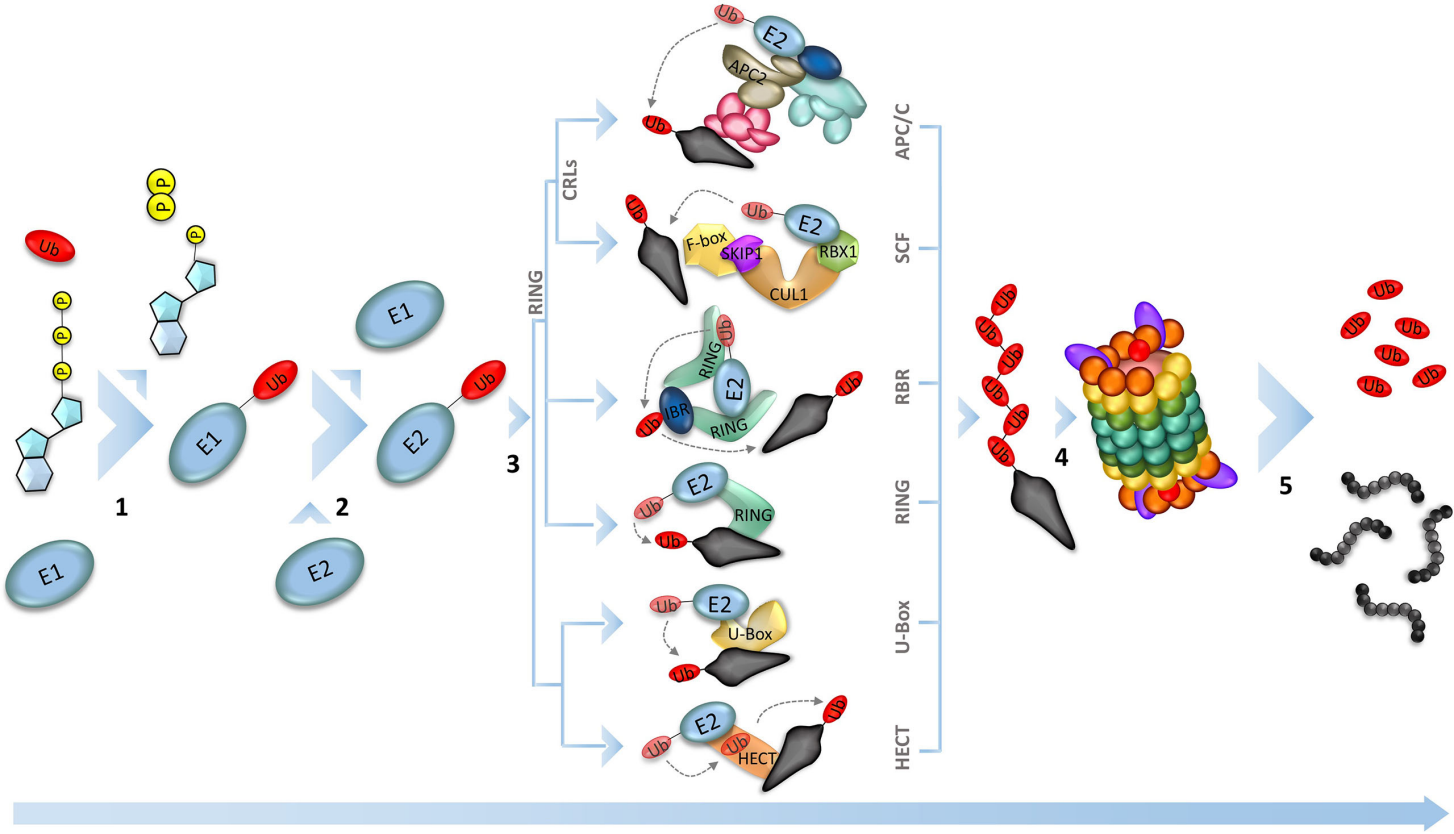
Ubiquitin-Proteasome System (UPS). The proteolysis in the UPS happens by sequential reactions catalyzed by three different enzymes. The process starts with the activation of the ubiquitin by the ubiquitin-activating enzyme (E1), using an ATP molecule (1). Next, the activated ubiquitin is transferred to the ubiquitin-conjugating enzyme (E2), which is responsible to interact with the ubiquitin ligase (E3) and conjugate the ubiquitin to the substrate that is recognized by the E3 (2). Plant genomes encode hundreds of E3 ligases (the main E3 ligases found in plants are represented in 3), that will target the different substrates, making them recognizable by the proteasome 26S, leading to the substrate degradation (5).

**Figure 2:**
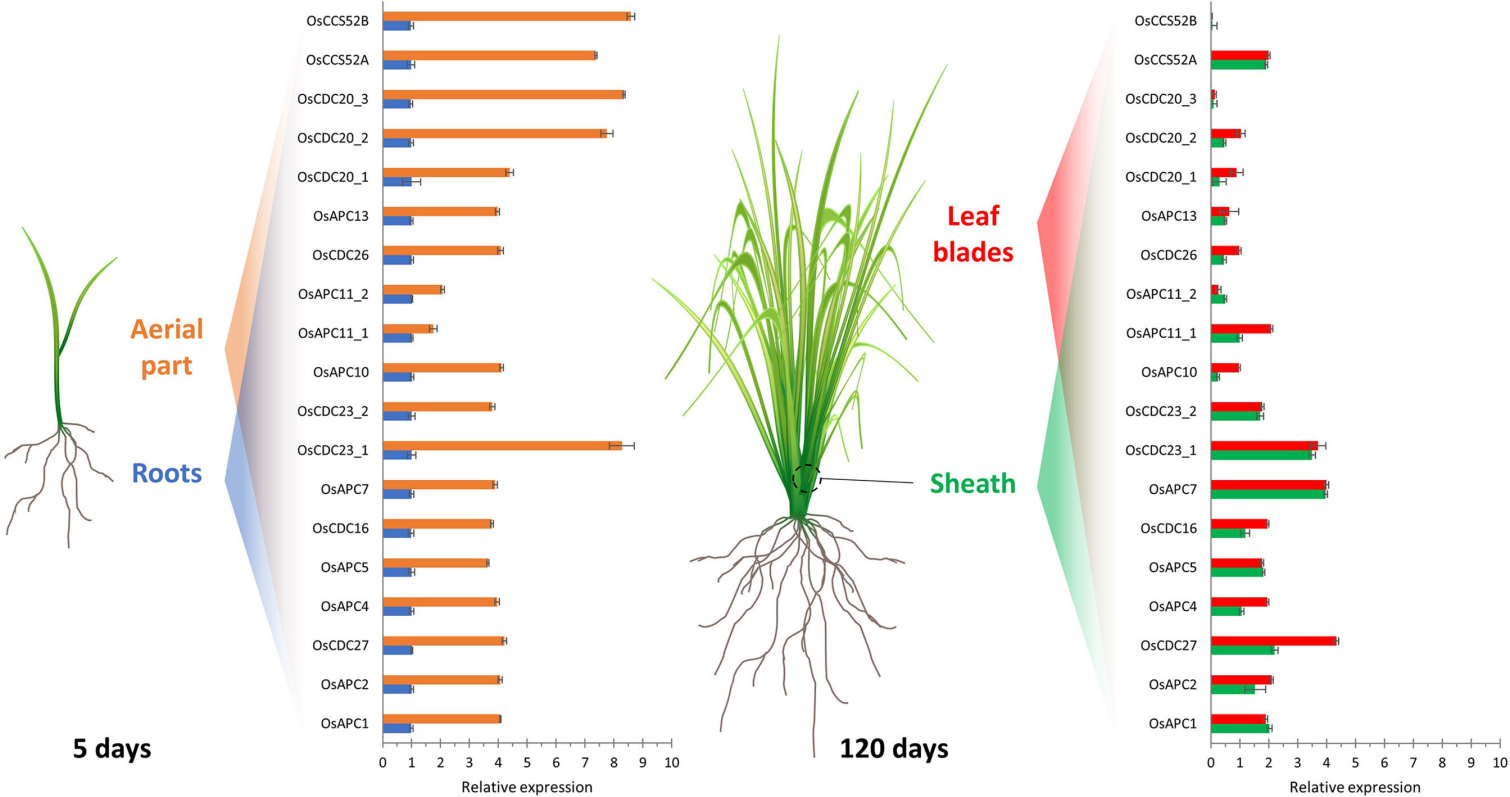
Relative expression profile of rice APC/C subunits and activators in different tissues. Expression analysis of genes from the APC/C subunits and activators in shoots and roots (5-day old seedlings) and in leaf sheath and blade (120-day old plants) of rice. Expression levels are normalized to root. Adapted from Lima et al., 2010.

To ensure a unidirectional progression of the cycle, cellular degradation mechanisms break down specific proteins that have phase-specific action (Genschik et al., 2014). In general, the ubiquitin-proteasome pathway is the main destruction machinery. This multi-enzymatic pathway adds a polyubiquitin tag on specific proteins, which will be recognized and degraded by the 26S proteasome (**Figure 2**) (Xu and Peng, 2006; Marshall and Vierstra, 2019). The importance of the ubiquitin-26S proteasome system (UPS) for plants can be exemplified by the high number of genes involved in this pathway in the *Arabidopsis thaliana* genome, covering approximately 6% of the total genes (Hua and Vierstra, 2011). The majority of those genes are responsible for the expression of E3-ligases, which are the most diverse component of the enzymatic cascade, necessary for the selective identification of the substrate to be marked for proteolysis (Smalle and Vierstra, 2004; Marrocco et al., 2010; Serrano et al., 2018);. This unbalance among the genes of the UPS enzymes can be illustrated by the number of those genes in rice (*Oryza sativa*). While the rice plant expresses 6 and 36 ubiquitin-activating enzyme (E1) and ubiquitin-conjugating enzyme (E2), respectively, the number of ubiquitin ligase enzyme (E3) genes exceeds 1100 (Al-Saharin et al., 2022). According to their catalytic domain, the isoforms of E3 ligases can be grouped into U-Box, HECT (homology to E6-associated carboxyl terminus), and RING (really interesting new gene). The U-box and HECT domains are mostly found in monomeric enzymes, but only the second one is known to form the E3-Ub intermediary (Wang et al., 2022). The RING-finger domain is found as a monomeric domain in a single subunit RING ubiquitin ligase and RBR (RING Between RING) ubiquitin ligase, which targets ABA receptors for degradation in different subcellular locations at root and leaves. Also, the RING domain is found in muti-subunit enzymes, such as Cullin RING Ligases (CRLs) domains (Fernandez et al., 2020; Wang et al., 2022). The E3s that have the RING domain are the most well characterized ligases in plants, remarkably the CRLs, once these enzymes play important role in plant growth and development (Chen and Hellmann, 2013; Serrano et al., 2018) (**Figure 1**). The Skp1/Cullin/F-box (SCF)-related complex and the Anaphase-Promoting Complex/Cyclosome (APC/C) are two well-characterized E3-ligases of the CRLs type in plant cell cycle control. It is already known that the SCF complex interacts with the D-type cyclins, forwarding them to degradation, while APC/C temporally removes the A- and B-type cyclins in the early-to-mid mitosis progression (Inzé and De Veylder, 2006).

As the name suggests, the APC/C is a key enzyme during anaphase initiation, allowing chromatid separation. Once activated, the APC/C can selectively target securin for degradation, which is an inhibitor of separase, an enzyme able to break up the cohesin complex that holds the chromatids together. The degradation of securin leads to separase activity and, consequently, the segregation of chromatids, marking the beginning of anaphase (Castro et al., 2005; de Lange et al., 2015; Jonak et al., 2017; Kernan et al., 2018). Securins are widely found in fungi and animals, but the presence of these proteins has not been detected in plants. However, Cromer et al. (2019) have reported two proteins, PATRONUS1 and PATRONUS2 (PANS1 and PANS2) in Arabidopsis, which would act similarly to securin in plants. The authors observed that APC/C is necessary for targeting PANS1 to trigger chromosome separation. Also, they showed that both proteins are essential to plant viability and can interact directly with SEPARASE (Cromer et al., 2019). Chromosome separation is the main reported function of the complex, but APC/C is also involved in the exit from mitosis and in the G1 phase of the cell cycle (Eytan et al., 2006; Alfieri et al., 2017). Since the discovery of the complex, 25 years ago, intensive studies have uncovered many aspects of APC/C regulation and its role in cell metabolism, but we are still far from a full understanding of this important cellular machinery, especially in monocot plants (Yamano, 2019).

The differential expression of APC/C subunits in various Arabidopsis tissues, even in completely differentiated cells, has driven research interest in understanding what other roles the complex can play in the organism (Lima et al., 2010; Eloy et al., 2011). In this review, we bring some of these additional functions performed by APC/C in different aspects of plant development and growth in monocotyledons of economic importance.

## APC/C is a ubiquitin ligase with multiple subunits

One of the most important mechanisms implicated in plant cellular and developmental processes is the post-translational regulation *via* ubiquitin-proteasome pathway/system (UPP/UPS) (Sharma et al., 2016; Stone, 2019). The UPP/UPS irreversibly conjugates ubiquitin moieties to the target proteins, resulting in polyubiquitylated proteins that will be recognized and degraded by the 26S proteasome, releasing the free ubiquitin for recycling (Miricescu et al., 2018; Marshall and Vierstra, 2019).

Protein ubiquitination is a multi-enzymatic cascade that involves successive activity of the enzymes that compose the UPS. The pathway starts with the E1-activating enzyme activating and transferring one ubiquitin to an E2-conjugating enzyme, in an ATP-dependent manner. Next, the E3 ubiquitin ligase enzyme mediates the transfer of ubiquitin from E2 to a lysine (Lys) residue into the target protein (Toma-Fukai and Shimizu, 2021). This labeling process is repeated several times because all seven Lys residues on the ubiquitin molecule are ubiquitinated. The polyubiquitylation of target proteins functions as a recognition motif for the large ATP-dependent multicatalytic protease (26S), the proteasome, which will subsequently degrade the polyubiquitinated proteins, using its endopeptidase activity, into small peptides (Smalle and Vierstra, 2004; Vierstra, 2009) (**Figure 2**).

E3 ubiquitin ligases comprise a large and diverse family among the three classes of enzymes involved in the ubiquitination proteolytic pathway. APC/C, an important conserved multi-subunit E3 ubiquitin ligase, is one of the most complex molecular machines known able to catalyze ubiquitination reactions. The complex mediates the degradation of several eukaryotic key cell cycle proteins, such as mitotic cyclins and securins (Petersen et al., 2000; Harper et al., 2002; Capron et al, 2003; Buschhorn and Peters, 2006). Besides its essentiality in cell cycle regulation, APC/C performs specific functions during plant development. Through functional characterization of their subunits, plant APC/C proteins have been reported to play a role during cell differentiation in shoot and root meristems (Blilou et al., 2002; Lin et al., 2020; Schwedersky et al., 2021), plant growth (Saze and Kakutani, 2007; Kuppusamy et al., 2009; Marrocco et al., 2009; Rojas et al., 2009; Kumar et al., 2010; Eloy et al., 2011; Eloy et al., 2012; de Freitas Lima et al., 2013), vascular development (Marrocco et al., 2009), hormone regulation (Blilou et al., 2002; Lin et al., 2020), tillering control (Lin et al., 2012; Xu et al., 2012; Lin et al., 2020), female and male gametogenesis (Capron et al., 2003; Kwee and Sundaresan, 2003; Eloy et al., 2011; Zheng et al., 2011; Wang et al., 2012), and embryogenesis (Pérez-Pérez et al., 2008; Awasthi et al., 2012; Wang et al., 2012; Wang et al., 2013; Guo et al., 2016). In plants, APC/C is composed of approximately 14 subunits, as seen in Arabidopsis, maize, and sorghum, which are divided into at least three main functional modules: a catalytic/substrate recognition module, including the APC2, APC11, and APC10; a structural module containing a tetratricopeptide repeat (TPR), formed by APC3, APC6, APC7, and APC8 (D’Andrea and Regan, 2003; Alfieri et al., 2017); and a scaffold module, to which the catalytic and structural components are attached, containing the APC1, APC4, and APC5 subunits (Thornton and Toczyski, 2003; Thornton et al., 2006; Schreiber et al., 2011; Chang et al., 2014; Chang et al., 2015; Eloy et al., 2015; Alfieri et al., 2017). APC13 and APC15 are accessory subunits responsible to promote the TPR association (Thornton et al., 2006; Chang et al., 2014; Chang et al., 2015; Alfieri et al., 2017). The CELL DIVISION CYCLE PROTEIN 26 (CDC26) subunit, recently identified as part of the APC/C, contains an upstream open reading frame (uORF) encoding a functional protein that may control the translation of the main ORF (mORF) (Lorenzo-Orts et al., 2019).

**Figure 3:**
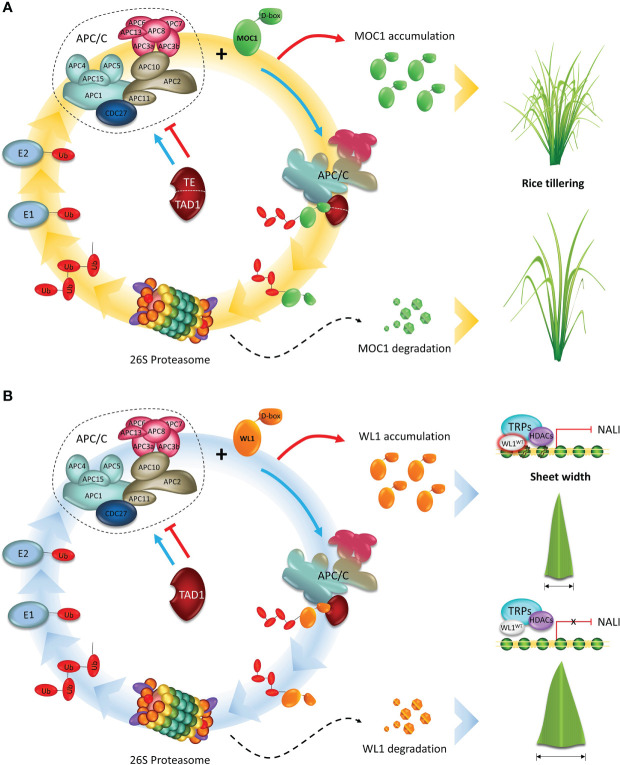
APC/C plays an essential role in regulating the development of cereal crops. **(A)** A model showing the APC/C^TE/TAD1^ complex-mediated degradation of MOC1. Tiller Enhance (TE)/Tillering and Dwarf 1 (TAD1) act as activators of the Anaphase Promoting Complex/Cyclosome (APC/C) complex E3 ubiquitin ligase activity and targets MOC1 for degradation through interacting with the D-box by the ubiquitin–26S proteasome pathway, and consequently represses tillering. **(B)** A proposed model for APC/C^TAD1-WL1-NAL1^ module-mediated control of leaf width. TAD1 activates the APC/C E3 ubiquitin ligase activity and targets WIDE-LEAF 1 (WL1) for degradation. WL1 directly binds to the regulatory region of NARROW LEAF 1 (NAL1) and recruits the corepressor TOPLESS-RELATED PROTEIN (TPR) to inhibit NAL1 expression by down-regulating the level of histone acetylation of chromatin, and consequently, decreasing leaf width. Adapted from Lin et al., 2012 and Xu et al., 2012.

Although most of the studies about APC/C have been carried out in budding yeasts (*Saccharomyces cerevisiae*), and in plants, have been carried out in the model *A. thaliana*, little is known about the complex in monocots. Homology-based sequence analysis showed that almost every Saccharomyces and Arabidopsis APC/C subunit are encoded by a single counterpart gene in monocots, except for *APC8* and *APC11* in rice, *APC6*, *APC8*, *APC10*, *APC11*, and *APC15* in maize; and *APC11* in sorghum (**Table 1**). Furthermore, in maize and sorghum, all APC/C subunits are present except for APC1 (Lima et al., 2010). In the rice genome, only a partial *APC1* sequence is present, possibly due to misannotation (Lima et al., 2010) (**Table 1**). These data suggest that ​monocots have all the necessary components to assemble a functional and active complex. Consequently, comparative genomic analyses can provide valuable insights into the organization of the cell cycle machinery and the evolution of these protein complexes, indicating that the mechanisms that drive APC/C-mediated proteolysis are conserved in organisms, including plants. Also, according to Lima et al. (2010), expression patterns can provide important clues for gene function under specific conditions. In rice, for example, the expression of several APC/C subunit genes has been investigated in roots and shoots of 5-day-old plants and in the sheath and blade of mature leaves (**Figure 3**). As expected, tissues with higher cell proliferation rates showed higher expression levels of APC/C genes, however, with a pattern varying from organ to organ (Lima et al., 2010). In general, the mRNA levels of *OsAPC1*, *OsAPC2*, *OsAPC4*, *OsAPC5*, *OsAPC10*, *OsAPC11_2*, *OsCDC26*, and *OsAPC13* are reduced in both sheath and blade compared to the total aerial part with 5 days old. Conversely, *OsAPC11_2* and *OsCDC27* mRNA levels are reduced only in the sheath but not in the blade. Finally, there is no reduction of *OsAPC7* expression in both sheath and blade. These results show that APC/C in monocots may have distinct characteristics that can be important for its function in this group, and the elucidation of these characteristics requires additional investigation.

**Table 1:** Genomic evolution of the Anaphase Promoting Complex/Cyclosome (APC/C).

	*A. thaliana*	*O. sativa*	*Z. mays*	*S. bicolor*	*S. cerevisiae*				
APC subunits	Access number					% identity*O. sativa*	% identity*Z. mays*	% identity*S.bicolor*	% identity*S.cerevisiae*
APC1	At5g05560		Zm00001d037981	Sobic.009G104900	KAF4007119.1		49	50	27
APC2	At2g04660	Os04g40830	Zm00001d014685	Sobic.010G252700	EGA73963.1	63	64	64	22
APC3a	At3g16320		Zm00001d042523	Sobic.003G388000	EEU08813.1		47	47	34
APC3b	At2g20000	Os06g41750	Zm00001d042523	Sobic.003G388000	EEU08879.2	55	57	56	34
APC4	At4g21530	Os02g54490	Zm00001d052072	Sobic.004G322300	EEU08879.142	49	52	52	29
APC5	At1g06590	Os12g43120	Zm00001d040342	Sobic.003G123100	NP_014892.3	53	51	50	30
APC6a	At1g78770	Os03g13370	Zm00001d028358	Sobic.001G443300	EEU08879.204	74	73	73	31
APC6b			Zm00001d047979				72		
APC7	At2g39090	Os05g05720	Zm00001d024858	Sobic.009G045000	–	62	61	61	
APC8a	At3g48150	Os02g43920	Zm00001d017475	Sobic.004G295900	EEU08879.256	65	66	66	30
APC8b		Os06g46540	Zm00001d042523			40	33		
APC10a	At2g18290	Os05g50360	Zm00001d009440	Sobic.009G217300	EEU08879.326	81	82	81	35
APC10b			Zm00001d038881				77		
APC11a	At3g05870	Os03g19059	Zm00001d028837	Sobic.001G398600	EEU08879.348	89	89	90	40
APC11b		Os07g22840	Zm00001d040164	Sobic.003G103400		88	37	37	
APC13	At1g73177	Os07g44004	Grmzm6g522911	Sobic.002G387800				53	
APC15a	AT5g63135	Os02g38029	Zm00001d050944	Sobic.004G200000		52	55	55	
APC15b			Zm00001d017070				53		
**Activators**									
CDC20_1a	At4g33260	Os09g06680	Zm00001d016034	Sobic.004G119700	EEU08879.448	71	70	72	46
CDC20_2	At4g33270	Os04g51110	Zm00001d000168	Sobic.004G270800	EEU08879.558	70	71	71	45
CDC20_3	At5g26900	Os02g47180			EEU08879.668	71			41
CDC20_4	At5g27080				EEU08879.779				44
CDC20_5	At5g27570				EEU08879.889				46
CDC20_6	At5g27945				EEU08879.1009				41
CCS52A1	At4g22910	Os03g03150	Zm00001d027430	Sobic.001G526300	EEU08879.1129	70	72	73	48
CCS52A1_2									
CCS52A2	At4g11920		Zm00001d048472		EEU08879.1237		60		50
CCS52B	At5g13840	Os01g74146	Zm00001d041957	Sobic.003G444100	EEU08879.1374	69	73	74	48

Two structurally related proteins act as co-activators of the APC/C, ensuring the complex activity. The CELL DIVISION CYCLE20 (CDC20) and the CDC20 HOMOLOG 1 (CDH1) are found in all known eukaryotic genomes (Peters, 2006). The APC/C co-activators are characterized by the WD-40 domain, tandem repeats termed after a high frequency of tryptophan (W) and aspartic acid (D) pairs, which represents the main site for protein interactions (van Leuken et al., 2008). The WD-40 class proteins are essential for providing catalytic activity and facilitating substrate recognition in APC/C-dependent proteolysis (van Leuken et al., 2008). The number of *CDC20* copies varies according to the species. In corn and sorghum, both genomes hold two copies (*CDC20.1* and *CDC20.2*), while the rice genome contains three copies (*CDC20.1*, *CDC20.2*, and *CDC20.3*) (**Table 1**). The plant CDH1 activators are known as CELL CYCLE SWITCH52 (CCS52) proteins (Peters, 2002; Baker et al., 2007; Breuer et al., 2012) and can be classified into A- and B- types, known as *CCS52A* and *CCS52B*, respectively (Cebolla et al., 1999; Tarayre et al., 2004; Kevei et al., 2011). The rice genome has two *CCS52* genes compared to three and two genes in maize and sorghum, respectively (**Table 1**). The overexpression of *OsCCS52A* in rice inhibits mitotic cell division and induces endoreduplication, also known as endocycling or endoreplication (detailed below), and cell elongation in fission yeast (Su'udi et al., 2012b). In addition, T-DNA insertion in the *OsCCS52A* resulted in rice plants with growth retardation and smaller seeds showing endosperm defects during endoreduplication. These phenotypes were attributed to disruption of the endoreduplication cycle in the endosperm of the mutant seeds, as evidenced by a reduction in nuclear and cell size (Su'udi et al., 2012b). Tillering and Dwarf mutant 1 (*tad1*), which is an ortholog of *CCS52A* (Xu et al., 2012), similarly caused semi-dwarfism and leaf size decrease in rice. Furthermore, the rice mutant line *osccs52b* exhibited a semi-dwarf and narrow kernel phenotype due to a reduction in cell expansion (Su'udi et al., 2012a). Microscopic analysis of mutant kernels showed that the nuclear size and ploidy level were unaffected. Together, these results suggest that *OsCCS52B* may be involved in cell expansion regulation in rice endosperm (Su'udi et al., 2012a).

Besides its activators, the APC/C is also controlled by inhibitory proteins, which are known in Arabidopsis as ULTRAVIOLET-B-INSENSITIVE4 (UVI4) and its homolog *OMISSION OF SECOND DIVISION 1 *(*OSD1)/GIGAS CELL 1 *(*GIGAS*)*/UVI4-Like* (Hase et al., 2006; d’Erfurth et al., 2009; Van Leene et al., 2010; Heyman et al., 2011; Iwata et al., 2011). The UVI4 and OSD1/GIGAS/UVI4-Like proteins are considered negative regulators of the APC/C activity and have a partially redundant action, since both can assemble to the CDC20, CCS52A, and CCS52B co-activator subunits. Likewise, the loss of *UVI4* and *OSD1/GIGAS/UVI4-Like* negatively affects the stability of the mitotic A-type cyclins (Heyman et al., 2011; Iwata et al., 2011; Cromer et al., 2012). In the Poaceae family, for example, an independent whole-genome duplication (Lloyd et al., 2014) led to two subgroups of OSD1/GIGAS/UVI4-Like genes and species of this family have, at least, one member of each subgroup in their genome (d’Erfurth et al., 2009). The rice genome contains two genes of OSD1/GIGAS/UVI4-Like (*Os02g37850* and *Os04g39670*), and it was shown that a single mutation in the *Os02g37850* was sufficient to give rise to the meiotic defects observed in the mutant plants, resembling the same phenotype observed in Arabidopsis *osd1* mutant (Mieulet et al., 2016). A single gene orthologous to *OsOSD1* was identified in barley and Brachypodium genomes, whereas the maize and sorghum genomes harbor a tandem duplication of the *OSD1* gene (Lloyd et al., 2014).

Another plant-specific regulator that directly interacts with APC/C is SAMBA. In Arabidopsis, SAMBA has been identified as a plant-specific regulator of the APC/C because its loss-of-function results in increased cell proliferation during early development, and biochemical analyses showed that the lack of SAMBA stabilizes CYCA2;3 (Eloy et al., 2012). Moreover, the endoreduplication rate of the *samba* mutant is higher, suggesting that, despite the increased cell number, cells exit the division cycle earlier.

In maize, CRISPR/Cas9 *samba* mutants also displayed higher cell proliferation, due to increased cell division rate with reduced cell size (Gong et al., 2022). However, despite the seemingly conserved role of SAMBA in associating with APC/C in maize (GRMZM2G157878), the phenotypic readout was distinct in Arabidopsis and maize plants. The *samba* mutants displayed dwarfism, erect upper leaves, reduced organ and tissue growth, which most likely results from several inter-species differences or a combination thereof (Gong et al., 2022). Moreover, it is noteworthy that a visible difference in *SAMBA* mRNA expression exists in Arabidopsis compared to maize. In Arabidopsis, the *SAMBA* transcript was higher during embryogenesis, decreased gradually when seedlings germinated, and is restricted to the hypocotyl at 8 days after stratification, while in maize, the expression of *SAMBA* is more stable throughout the entire plant life cycle (Sekhon et al., 2011).

## APC/C plays an essential role in seed shape and size in cereal crops

Cereals are the main class of crops in the world supplying a substantial portion of food and industrial raw materials to mankind (Olsen, 2020). Mature cereal grains characteristically contain three major structures: embryo, endosperm and/or embryonic cotyledons, and seed coat. The endosperm accounts for most of the seed’s volume. Its shape and size are highly determined by cell size through growth and expansion (Kobayashi, 2019), as well as by a large accumulation of storage compounds, like carbohydrates, proteins, and/or lipids, and water (Dante et al., 2014a; Hands et al., 2016). Grass endosperm development has several distinct phases, which can overlap considerably, such as early development, differentiation, periods of mitosis and later endoreduplication, accumulation of storage compounds, and maturation (Sabelli and Larkins, 2009).

The endoreduplication process has been extensively described in monocot species (Sabelli and Larkins, 2009), displaying a huge impact on their cell’s ploidy level (Dante et al., 2014b). The endoreduplication cycle occurs during the transition from the mitotic cell cycle to a modified cycle called endocycle, during which DNA re-replication is stimulated without subsequent chromosome segregation and cytokinesis (Kobayashi, 2019). In this process, the chromatids are duplicated exponentially, while the number of chromosomes remains unchanged (Edgar and Orr-Weaver, 2001). Endoreduplication is an integral part of plant development. This process is observed in different cell types, however, it is more prominent in larger, metabolically active, or highly specialized cells (Inze and De Veylder, 2006; De Veylder et al., 2011) as the ones forming the endosperm of Poaceae seeds (Sabelli and Larkins, 2009; Sabelli, 2012). The prevalence of endoreduplication in cereal grains suggests that it might have been positively selected during plant domestication and breeding (Nowicka et al., 2021). During endoreduplication, as cells expand, metabolic products, such as starch and storage proteins, are accumulated in the seed endosperm (Sabelli et al., 2013). The peak of endoreduplication events during the endosperm development occurs 15 days after planting (DAP) (Sabelli and Larkins, 2009). Nowicka et al. (2021), by using different barley cultivars, showed a natural variation in the kinetics of this process. These cultivars have a high degree of endoreduplication in endosperm during the second half of the barley grain growth period, characterized by the production of storage components (Dante et al., 2014a). The major wave of endoreduplication started from ~6 DAP and increased linearly to 20 DAP. In maize endosperm, this major wave occurs at 12 to 14 DAP, while it peaked at 15–18 DAP (Brian et al., 2001), and at 15~24 DAP in wheat (Sabelli and Larkins, 2009).

Endoreduplication can influence cereal grain yield and quality. For instance, the frequency of polyploidy and the number of cells per endosperm are correlated with seed weight in wheat (Brunori et al., 1993). The phenotypic and molecular consequences of endoreduplication in endosperm remain unclear and seem to be species dependent (Nowicka et al., 2021). The onset of endoreplication occurs when CDK/Cyclin complex is low or inactive (Lilly and Duronio, 2005; Inze and De Veylder, 2006), which is often associated with the degradation of mitotic cyclins by the APC/C and their activators (Cebolla et al., 1999). In maize endosperm, induced S-phase CDK activity and repressed M-phase CDK activity were proposed to trigger endoreduplication cycles (Grafi and Larkins, 1995). Dante et al. (2014b), when studying the expression patterns of some cell cycle proteins like A-, B- and D-type cyclins, and A- and B-type CDKs, as well as their kinase activity, demonstrated that CYCA1-associated kinase activity was higher during the mitotic stage of endosperm development. In contrast, CYCB1;3, CYCB2;1, and CYCD5-associated kinase show higher activity in the mitosis-to-endoreduplication transition. Furthermore, A-, B-, and D-type cyclins were more resistant to proteasome-dependent degradation in endoreduplicating endosperm extracts compared to mitotic extracts. Taken together, these results suggest that endoreduplication is associated with reduced cyclin proteolysis through the ubiquitin-proteasome pathway (Dante et al., 2014b).

CCS52 protein has also been reported to be involved in endoreduplication in seeds (Larson-Rabin et al., 2009; Mathieu-Rivet et al., 2010), however, collectively, OsCCS52A and OsCCS52B seem in part distinct from their dicotyledon orthologs. The important cell cycle regulator gene *CCS52A*, by activating the APC/C, is responsible for the mitotic-endocycle transition and modifications in its expression levels hamper the endosperm development (Barrôco et al., 2006; Su'udi et al., 2012a). To investigate the functional role of the *OsCCS52A* during rice development, the T-DNA-insertional mutagenesis approach was used (Su'udi et al., 2012b). The *osccs52a* mutants exhibited smaller seeds and poorly developed endosperm as a result of decreased cell and nucleus sizes. Thus, *OsCCS52A* was also confirmed to play an important role during vegetative growth in rice plants, as well as being involved in the endoreduplication process during endosperm development. Moreover, reduced expression of the *OsCCS52B* gene in rice plants negatively impacted seed and cell size. However, no visible effect was observed during the endoreduplication cycle (Su'udi et al., 2012a).

## The APC/C regulation in cereal crops

In addition to its essential role in the cell cycle progression, the APC/C has also been reported to target different substrates in non-proliferating cells, such as *MONOCULM 1* (*MOC1*) gene, identified as a key regulator of rice tillering and branching control (Li et al., 2003). *MOC1* encodes a transcriptional regulator belonging to the GRAS (GAI, RGA, and SCR) family (Pysh et al., 1999), and it is mainly expressed in the axillary buds, promoting the initiation of the axillary buds and boosting their outgrowth during the vegetative and reproductive stages. The rice *moc1* full knockout mutants are characterized by having a single main culm without any tillers and reduced panicle branches (Lin et al., 2012; Xu et al., 2012). In wheat, the *TaMOC1* gene, ortholog of rice *MOC1*, is a typical nuclear protein with transcriptional activation motifs mainly involved in spikelet development (Zhang et al., 2015). These observations suggest that gene function is broadly conserved between species but the phenotypic changes and developmental effects are species-specific (Wang et al., 2018).

Moreover, two genes, Tillering and Dwarf 1 (*TAD1*) and Tiller Enhance (*TE*), were identified by co-expression analysis with *MOC1* in the axil leaves, ensuring rice tillering and branching control (**Figures 3A, B**). To perform the analysis, the authors worked with rice plants from a mutant pool, originated by self-crossing of a diploid plant from an autotetraploid culture. *TAD1* gene (Xu et al., 2012) was identified and isolated from the *tad1* mutant, which showed an increased tiller number, reduced plant height, and twisted leaves and panicles. Sequence analysis revealed that this mutation was caused by a single base substitution at the second exon of *tad1* resulting in G to A change, which produces a premature stop codon.

The *te* mutant (Lin et al., 2012), displayed a drastically increased tiller number and a twisted flag leaf. Through *in vitro* and *in vivo* interactions studies, the TAD1 and TE (Lin et al., 2012; Xu et al., 2012) were classified as CCS52 orthologs in rice. Sequence and phylogenetic analyses revealed that TAD1 and TE contain several conserved domains frequently found in other Cdh1 homologs, including WD-40 repeats domain and four motifs: CSM (Cdh1-specific motif), IR (APC binding domain), CBM (mitotic RVL cyclin binding motif), and RVL (mitotic cyclin binding motif). TAD1 and TE play an essential role during MOC1 degradation *via* APC/C, which results in the inhibition of tillering in rice. According to Xu et al. (2012), *TAD1*-overexpressing plants showed a reduced tiller number, which resembles the *moc1* phenotype. Furthermore, TAD1 interacts with MOC1 by coimmunoprecipitation and bimolecular fluorescence complementation (BiFC) assays, forming a complex with OsAPC10 and acting as a co-activator of APC/C to target MOC1 for degradation in a cell-cycle-dependent manner. In the absence of TAD1 function, MOC1 fails to be recruited for the APC/C dependent degradation, resulting in an accumulation of endogenous MOC1 proteins, and thus increasing the tiller number in *tad1* rice plants.TE is a substrate-recognition and binding factor of the APC/C, forming the APC/C^TE^ complex and interacting with MOC1 and OsCDC27 (Lin et al., 2012).

Rice *te* loss-of-function mutants exhibited increased and reduced sensitivity to abscisic acid (ABA) and gibberellic acid (GA) hormones, respectively (Lin et al., 2015). Both BiFC and *Co*-*immunoprecipitation (*Co-IP) assays showed that TE physically interacts with ABA receptors OsPYL (PYR1-LIKE)/RCARs (REGULATORY COMPONENTS OF ABA RECEPTORS) and promotes their degradation *via* proteasome 26S, repressing ABA signaling. Conversely, ABA inhibits APC/C-^TE^ activity by phosphorylating TE through activating the Sucrose Non-Fermenting-1-Re*lated Protein Kinase* 2 (SnRK2s), which may interrupt the interaction of TE to its substrates and subsequently stabilize OsPYL/RCARs. In contrast, GA3 treatment reduced the accumulation of SnRK2 proteins and may promote APC/C^TE^-mediated degradation of OsPYL/RCARs. Based on these data, it was proposed that SnRK2-APC/C^TE^ regulatory module represents a regulatory hub underlying the antagonistic action of GA and ABA in plants (Lin et al., 2015; Lin et al., 2020).

More newly, biochemical and genetic analyses revealed that TAD1, WL1 (WIDE-LEAF 1), and NAL1 (NARROW LEAF 1) function in a common pathway to control leaf width in rice (You et al., 2022) (**Figures 3A, B**). *WL1* gene was identified and isolated from the *wl1* mutant, which showed an increased leaf width throughout the growing season. Sequence analysis showed a single-nucleotide substitution from C to T was identified in the annotated gene. In resume, WL1 protein was able to bind to the regulatory region of NAL1 directly and then recruit the corepressor TOPLESS-RELATED PROTEIN (TPR) to inhibit NAL1 expression by regulating the level of histone acetylation of chromatin. In *wl1* rice plants, WL1 interacts with TAD1 and activates the APC/C^TAD1^, which targets WL1 for degradation, resulting in the decrease of endogenous WL1 proteins, and thus increasing leaf width. Thus, these discoveries uncovered a new mechanism underlying shoot branching and leaf width, and shed light on the understanding of how the cell-cycle machinery regulates plant architecture in monocots.

## Perspectives

Results from our research group suggest that the proteins forming the body of the APC, and others that interact with the complex, play a key role during proliferation in plants, leading to higher biomass (Rojas et al., 2009; Eloy et al., 2011; Eloy et al., 2012). Moreover, several studies have identified numerous proteins, such as hormone regulators, transcription factors, cell-division regulators, and cell wall biosynthetic proteins, as potential candidates for biomass enhancement (Cockcroft et al., 2000; Biemelt et al., 2004; Matsumoto-Kitano et al., 2008; Fornalé et al., 2012; Shen et al., 2012). Thus, engineered cereal crops with increased biomass are an excellent resource to overcome problems like adverse impacts of climate change, food shortage, and fossil fuel dependency.

A central goal of crop deployment is to develop varieties that meet our growing demands for better fitness and yield. Despite the great contribution of conventional breeding to this field, it is still necessary to develop new biotechnological tools such as CRISPR to produce novel cereal cultivars exhibiting better traits without compromising plant productivity.

The use of genetically modified organisms or the identification of compounds with positive effects on plant growth may increase the supply of biomass for different purposes and accelerate classical breeding approaches to ensure future crop productivity.

## Author contributions

NE conceived the manuscript. PO and LS wrote and NE revised and corrected the article. PO and LS prepared the figures. All authors contributed to the article and approved the submitted version.

## Funding

This research was supported by the São Paulo Research Foundation (FAPESP), NBE 2017/10333-8, PNO 2021/06611-8, and LFCS 2021/03212-5.

## Conflict of interest

The authors declare that the research was conducted in the absence of any commercial or financial relationships that could be construed as a potential conflict of interest.

## Publisher’s note

All claims expressed in this article are solely those of the authors and do not necessarily represent those of their affiliated organizations, or those of the publisher, the editors and the reviewers. Any product that may be evaluated in this article, or claim that may be made by its manufacturer, is not guaranteed or endorsed by the publisher.
